# Identification of An mtDNA Setpoint Associated with Highest Levels of CD44 Positivity and Chemoresistance in HGC-27 and MKN-45 Gastric Cancer Cell Lines

**DOI:** 10.22074/cellj.2018.5309

**Published:** 2018-05-28

**Authors:** Gökhan Terzioğlu, Özlem Türksoy, Ömer Faruk Bayrak

**Affiliations:** 1 Department of Biotechnology, Yeditepe University, Inönü Mahallesi, Kayışdağı Cad. 326A 26 Ağustos Yerleşimi, 34755 Ataşehir-İstanbul, Turkey; 2 Department of Medical Genetics, Faculty of Medicine, Yeditepe University, Inönü Mahallesi, Kayışdağı Cad. 326A 26 Ağustos Yerleşimi, 34755 Ataşehir-İstanbul, Turkey

**Keywords:** Antineoplastic Drug Resistance, Gastric Cancer, Mitochondria, Mitochondrial DNA

## Abstract

**Objective:**

Cancer stem cells (CSCs) have important roles in survival and chemoresistance. These cells are commonly recognized with CD44 and CD24 markers. In this study, we aimed to analyze the effects of mtDNA content on cell surface positivity for anti-CD24 and anti-CD44 antibodies and chemoresistance level in AGS, HGC-27 and MKN-45 gastric cancer (GC) cell lines and to determine a setpoint for mtDNA copy for each cell line.

**Materials and Methods:**

In this experimental study, we initially decreased mtDNA levels in AGS, HGC-27 and MKN-45 by EtBr treatment. This depletion was confirmed with quantitative polymerase chain reaction (qPCR). Changes in cell surface positivity for anti-CD24 and anti-CD44 antibodies in control and mtDNA-depleted AGS, HGC-27 and MKN-45 were then analyzed with flow cytometry. Changes in chemoresistance (5-FU and cisplatin) were analyzed for all cell lines. The relationship between mtDNA content and cell surface positivity for CD24 and CD44 markers was examined.

**Results:**

The highest CD44 positivity was found in HGC-27 and MKN-45 ρlow cells which had 33-40% mtDNA content of control cells, however, CD24 positivity decreased with mtDNA depletion in all cell lines. The highest chemoresistance levels were found in all ρlow cells. mtDNA-recovered (i.e. reverted) HGC-27 and MKN-45 cells partially maintained their increased chemoresistance while reverted AGS cells did not maintain an increased level of chemoresistance.

**Conclusion:**

mtDNA depletion triggers chemoresistance in cancer cell lines and is correlated with increase and decrease of CD44 and CD24 positivity respectively in HGC-27 and MKN-45 GC cell lines. A mtDNA content above or below the identified setpoint (33-40% of that in control cells), results in the decrease of CD44 positivity and chemoresistance levels.

## Introduction

In recent years, there has been progress in treating gastric cancer (GC) with the widespread use of new surgical techniques for tumor resection and lymph node dissection. With the development of adjuvant chemotherapy and targeted molecular therapies, condition of patients have improved significantly (1). Although the incidence of GC has declined in recent years, it still remains the fifth most common cancer in the world. Patients with inoperable, metastatic or recurrent disease have very low survival rate, even after palliative cytotoxic chemotherapy (2). 

Cancer stem cells (CSCs), which have roles in survival and chemoresistance, are commonly analyzed according to the expression of CD44 and CD24 markers (3). CD44 is a cell surface glycoprotein and an adhesion molecule which provides signal transduction through cell-cell communication. CD44 has several functions in migration, adhesion and signalization (4). The expression of CD44 was found to be correlated with survival, tumor size, stage and metastasis in GC (5). CD44 is also a GC stem cell marker and not only CD44+ GC cells were found to be chemoresistant, but the expression level of CD44 is associated with the onset and progression of gastric tumors (6, 7).

CD24, a cell surface protein linked to glycosylphosphotidyl- inositol, is a heat-stable antigen which is heavily glycosylated and involved in cell-cell and cell- matrix interactions (8). CD24 overexpression can inhibit an anti-apoptotic signaling pathway in CD44+ tumor cells and accelerate apoptosis as an answer to DNA damage (9). CD24 is also an important diagnostic and prognostic marker of cancer given its expression in many tumor types. In some types of cancer, such as breast cancer, CSCs have decreased CD24 expression (10). However, in certain tumor types, such as nasopharyngeal, it has been suggested as a CSCs marker (11). Accordingly, the status of CD24 as a CSC marker remains vague when compared with CD44. 

mtDNA depletion is a common event in GC, which may induce CD44 expression in cancer cells (12-14). This depletion has been shown to induce the generation of CSCs, invasion and metastasis, and expression of epithelial-mesenchymal transition (EMT) markers. In addition, it promotes pro-survival and anti-apoptotic pathways which may lead to chemoresistance (13, 15, 16). In hepatocellular carcinoma and breast cancer, increased expression of antioxidant enzymes such as glutathione peroxidase and manganese superoxide dismutase has been observed, which may increase chemoresistance via altered redox-antioxidant regulation (17-19). Although it is known that mtDNAdepletion increases chemoresistance (20) and CD44 positivity (13) in cancer cells, the level of mtDNA depletion that causes the greatest increase in chemoresistance and CD44 expression as a setpoint has not yet been determined. 

We therefore aimed to analyze the effects of mtDNA content on cell surface positivity for CD44 and CD24, and chemoresistance (5-FU and cisplatin) in AGS, HGC-27 and MKN-45 GC cell lines. We show that the observed setpoint of mtDNA level results in the highest CD44 positivity (as a CSC marker) and chemoresistance to both 5-FU and cisplatin. 

## Materials and Methods

In this experimental study, AGS (a non-metastatic GC cell line derived from poorly differentiated gastric adenocarcinoma), HGC-27 (derived from lymph node metastasis of GC) and MKN-45 (metastatic gastric cancer cell line derived from a poorly differentiated gastric adenocarcinoma) were cultured in appropriate media (2123). HGC-27 and MKN-45 were cultured in RPMI-1640 (Gibco, USA) containing 10% fetal bovine serum (FBS, Gibco, USA) while AGS was cultured in DMEM-F12 (Gibco, USA) containing 10% FBS. All culture media contained 50 µg/ml uridine (U6381, Sigma-Aldrich, USA) and 1 mM sodium pyruvate (P2256, Sigma-Aldrich, USA). All cells were cultured at 37°C in a humidified atmosphere with 5% CO_2_ in air.

### mtDNA depletion and reversion 

mtDNA levels of AGS, HGC-27 and MKN-45 were reduced with low dose ethidium bromide (EtBr, 50 ng/ml) treatment in the presence of 50 µg/ml uridine and 1mM sodium pyruvate.

Varying levels of mtDNA depletion were applied on HGC-27 and MKN-45 cells to identify the mtDNA setpoint at which the highest cell surface positivity for anti-CD44 antibody was obtained. Given that mtDNA depletion decreased cell surface positivity for anti-CD44 antibody in AGS cells, changes in CD44 positivity were not analyzed with respect to different mtDNA levels. 

To revert ρ^low^ cells, they were transferred to a EtBr-free culture medium and remained in this medium until the cells gained approximately normal mtDNA levels (>94% of mtDNA levels of control cells). The resulting cells are referred to here in after as 'reverted'. 

### Analysis of mtDNA copy number 

Total DNA was isolated with Qiagen DNA Mini Kit (Qiagen, Germany). Relative changes in mtDNA copy number were analyzed with quantitative polymerase chain reaction (qPCR). The mtDNA-encoding mitochondrial NADH dehydrogenase(*MT-ND1*) gene specific primers (Integrated DNATechnologies, USA) and *Universal* Probe Library (UPL) probes (Roche, USA) were used for the analysis of changes in mtDNA copynumber. The nuclear DNA-encoding beta globin (*HBB*) genespecific primers (Integrated DNATechnologies, USA) and UPLprobe (Roche, USA) were used for normalization of expression changes since each cell has twoand multiple copies of nuclear and mitochondrial genomes respectively and this may thus beused for normalizing data. The primers and probes that are usedin for this test are shown in Table 1. For all qPCR reactions, FastStart Universal Master Mix (Roche, USA) and the Roche Light Cycler 480 instrument (Roche, USA) were used. 

**Table 1:** Primers and probes used in the analysis of mtDNA copy number


Gene name	Sequence primer (5ˊ-3ˊ)	Probe and catalog number

*HBB*	F: TTTTGCTAATCATGTTCATACCTCTT	UPL probe #61-04688597001
	R: CCAGCACACAGACCAGCA	
*MT-ND1*	F: AACCTCTCCACCCTTATCACAA	UPL probe #51-04688481001
	R: TCATATTATGGCCAAGGGTCA	


### Flow Cytometry 

For flow cytometric analysis, trypsinized cells were washed twice with phosphate-buffered saline (PBS). Cell pellets were then resuspended and stained with CD44 (Biolegend, USA) and CD24 (BD Pharmingen, USA) antibodies. Gates were adjusted according to the unstained samples. All analyses were run on a BD FACS Aria III instrument (Becton Dickinson, USA). 

### Chemosensitivity assay

Cells were seeded in 96 well plates at a density of 5000cells/well in 150 µl of medium or without (i.e. control) chemotherapeutic drugs [fluorouracil (5-FU) and cisplatin] intriplicate. For the chemosensitivity assay, cells were treatedwith 1-1.5 µg/ml5-FU and 0.5-0.75 µg/ml cisplatin for 48hours. The MTS assay was then used to assess the relativeviability of cells. CellTiter 96® AQueous One SolutionReagent (Promega, USA) was added to each well and plateswere incubated at 37°C for 2 hours immediately after thechemotherapeutic treatment. Cell viability was assessed bymeasuring absorbance at 490 nm with the ELx800 ELISA microplate reader (BioTek, USA).

### Statistical analysis

Each experiment was performed in triplicate. One-way ANOVA with post-hoc Tukey HSD was used to test for differences among AGS, MKN-45 and HGC-27 cell lines. P<0.05 was considered as statistically significant.

## Results

### Identification of mtDNA setpoint for the highest CD44 positivity

**Fig.1:**
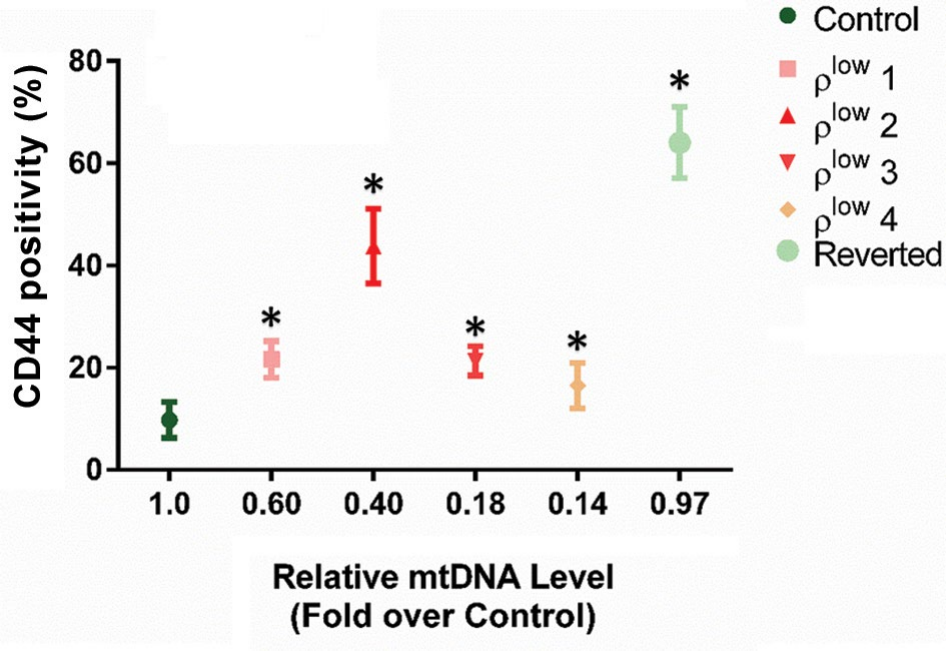
Changes in CD44 positivity with respect to mtDNA content in HGC-27 cells. Error bars represent SD. Asterisks (*) indicate statistical significance (P<0.05).

We measured CD44 levels corresponding to different mtDNA content. CD44 positivity reached its maximum valueA when the mtDNA level was at 33-40% of that observed in control cells of HGC-27 and MKN-45 cells (P<0.05). The changes in CD44 positivity with respect to mtDNA content for HGC-27 cells (Fig .1). A similar trend in CD44 positivity was also observed for MKN-45 cells (data not shown because the changes in cell surface positivity to CD44 in MKN-45 cells were slight and in the range of 1-2%). HGC-27 cells were only shown in Figure 1. In contrast, mtDNA depletion B decreased CD44 positivity in AGS cells and the changes in CD44 positivity were not analyzed with respect to different mtDNA levels. Therefore, AGS cells with 33-40% mtDNA content of control cells were used as ρ^low^ AGS cells. 

### mtDNA setpoint effect on CD44 positivity 

**Fig.2:**
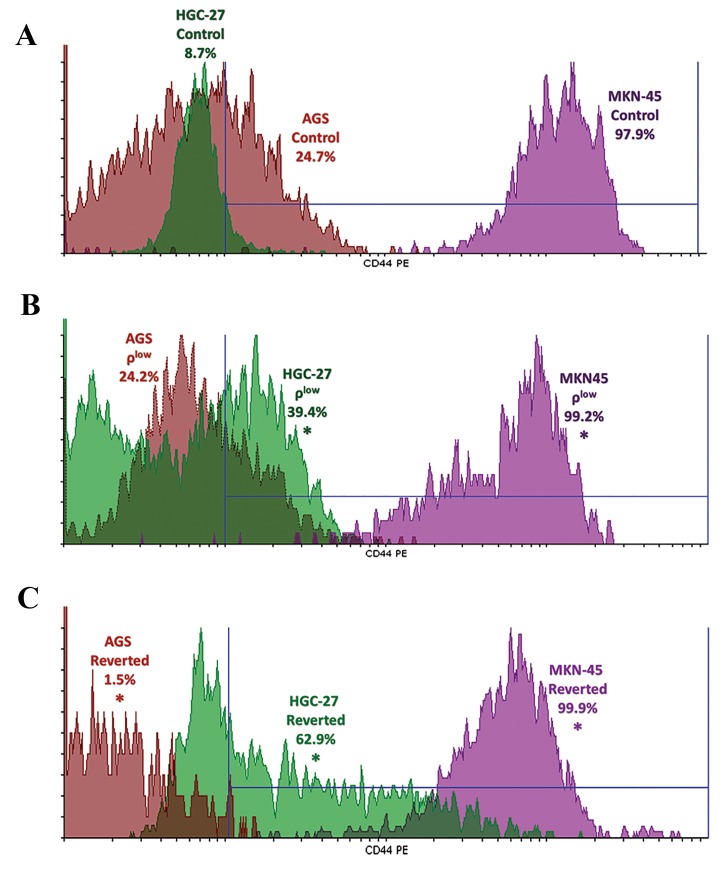
The cell surface positivity foranti-CD44 antibody. A. The overlay histogram of anti-CD44 antibody staining in AGS, HGC-27 and MKN-45 control cells, B. The overlay histogram of anti-CD44 antibody staining in AGS, HGC-27 and MKN-45 ρlow cells, and C. The overlay histogram of anti-CD44 antibody staining in AGS, HGC-27 and MKN-45 reverted cells. Asterisks (*) show statistical significance (P<0.05) based on comparison with controls of each cell line.

Depletion of mtDNA to the identified setpoint increased CD44 positivity in both HGC-27 (350% increase over control cells) and MKN-45 cells (1% increase over control cells) (P<0.05), however, mean fluorescence intensity (MFI) levels were increased only in HGC-27 ρ^low^ B cells. For HGC-27 (620% increase over control cells) and MKN-45 (2% increase over control cells), the increase in positivity and the MFI levels of CD44 remained after the cells were reverted (P<0.05). The overlay histograms of CD44 positivity for control, ρ^low^ and reverted cells (Fig .2). As expected mtDNA depletion to the setpoint also decreased cell surface positivity to anti-CD44 antibody in AGS cells, however, this decrease (2%) was minimal.

### mtDNA depletion decreased CD24 Positivity in AGS, HGC-27 and MKN-45 cells 

**Fig.3:**
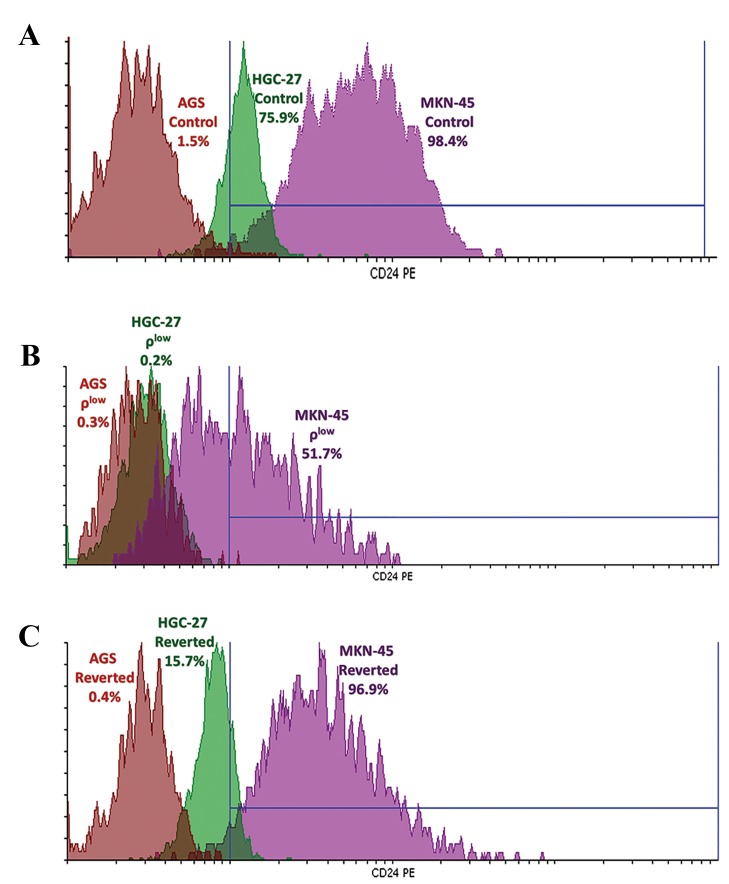
The cell surface positivity foranti-CD24 antibody in AGS, HGC-27 and MKN-45 cell lines. A. The overlay histogram of anti-CD24 antibody staining in AGS, HGC-27 and MKN-45 control cells, B. The overlay histogram of anti-CD24 antibody staining in AGS, HGC-27 and MKN-45 ρlow cells, and C. The overlay histogram of anti-CD24 antibody staining in AGS, HGC-27 and MKN-45 reverted cells. An asterisk (*) shows statistical significance (P<0.05) based on comparison with controls of each cell line.

Among the control cells, the highest CD24 positivity was found in MKN-45 cells and the lowest in AGS cells, showing very low levels. After depletion of mtDNA to the setpoint, CD24 positivity was reduced in all AGS (80% decrease over control cells), HGC-27 (nearly 100% decrease over control cells) and MKN-45 (48% decrease over control cells) cells. Unlike reverted AGS, reverted HGC-27 and MKN-45 partially regained cell surface positivity for CD24 (Fig .3). 

### Effect of the mtDNA setpoint on chemoresistance 

**Fig.4:**
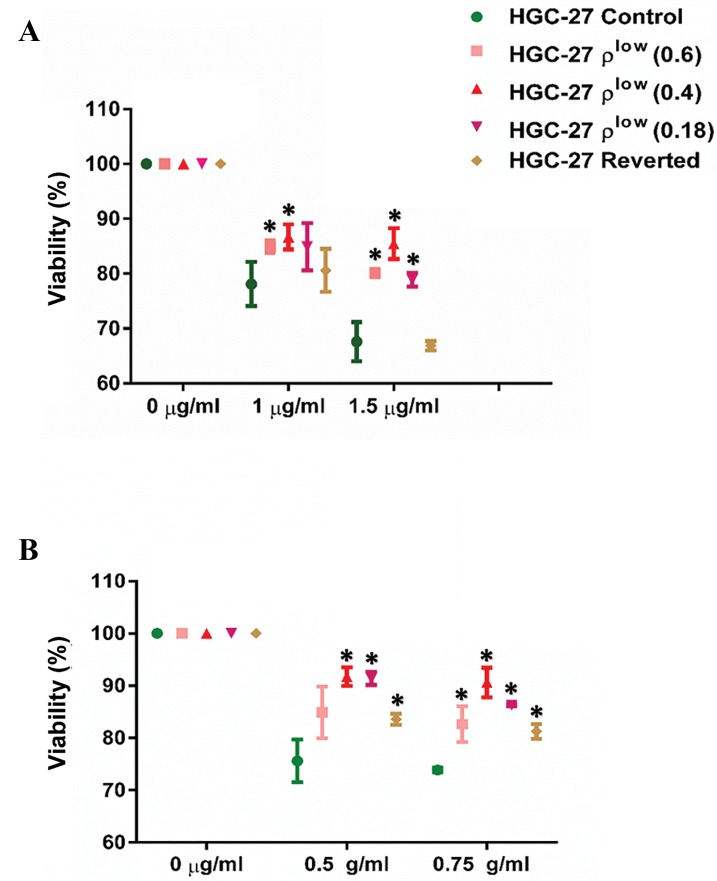
Changes in chemoresistance for control, ρ^low^ (0.6, 0.4, 0.18) and reverted HGC-27 cells. A. 5-FU and B. Cisplatin. Error bars represent SD. Asterisks (*) indicate statistical significance (P<0.05).

At the first step of chemoresistance analysis, changes in chemoresistance levels were analyzed for HGC27 cells with different mtDNA contents to identify the potential correlation with changes in anti-CD44 antibody positivity. HGC-27 was selected for this analysis since changes in CD44 positivity with respect to mtDNA depletion was most strongly associated in this cell line. The highest chemoresistance was found for HGC-27 ρ^low^ cells with mtDNA levels at the setpoint (P<0.05). The changes in chemoresistance to 5-FU and cisplatin with respect to mtDNA levels in HGC-27 (Fig .4). 

The mtDNA setpoint increased 5-FU and cisplatin chemoresistance of AGS, HGC-27 and MKN-45 ρ^low^ cells while the most prominent was observed in mtDNA-depleted AGS cells. AGS cells (88% increase for 1 µg/ml 5-FU, 100% increase for 1.5 µg/ml 5-FU, 5% increase for 0.5 µg/ml cisplatin and 11% increase for 0.75 µg/ml), HGC-27ρ^low^ cells (11% increase for 1µg/ml 5-FU, 19% increase for 1.5 µg/ml 5-FU, 8% increase for 0.5 µg/ml cisplatin, 10% increase for 0.75 µg/ml cisplatin) (P<0.05) and MKN-45ρ^low^ cells (35% increase for 1 µg/ml 5-FU, 50% increase for 1.5 µg/ ml 5-FU, 12% increase for 0.5 µg/ml cisplatin, 46% increase for 0.75 µg/ml) (P<0.05).

**Fig.5:**
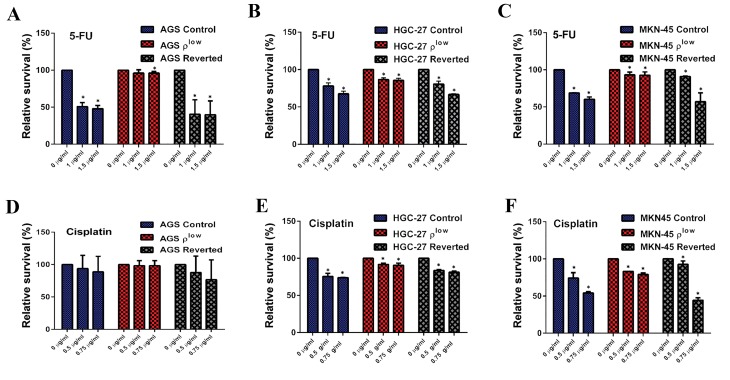
Changes in chemoresistance for 5-FU and cisplatin in AGS, HGC-27 and MKN-45 cells. A-C. Changes in chemoresistance for 5-FU in control, ρ^low^ (33-40%) and reverted AGS, HGC-27 and MKN-45 cells, respectively and D-F. Changes in chemoresistance for cisplatin in control, ρ^low^ and reverted AGS, HGC-27, MKN-45 cells, respectively. Error bars represent SD. Asterisks (*) indicate statistical significance (P<0.05).

After the mtDNA content was returned to normal levels, chemoresistance remained for low doses of 5-FU and cisplatin in reverted HGC-27 and MKN-45 cells. However, in AGS cells, chemoresistance was lower in reverted cells than in control cells (Fig .5).

## Discussion

In this study, a mtDNA setpoint was identified for the highest levels of chemoresistance and CD44 expression (as CSC marker) in GC cell lines. We observed the highest levels of cell surface positivity for CD44 (for HGC-27 and MKN-45 cells) and chemoresistance (for all three cell lines) when mtDNA content is depleted to 33-40% of that in control cells. Interestingly, the levels of chemoresistance and cell surface positivity for CD44 decreased when mtDNA depletion was either above or below this level. Some previous studies analysed the effect of mtDNA depletion on chemoresistance and CD44 expression in cancer cells but they failed to identify the mtDNA setpoint (12-14, 20).

It has been indicated that CD44 is a chemoresistance inducer (24-26). The changes in cell surface positivity for anti-CD44 antibody were correlated with changes in chemoresistance levels of metastatic HGC-27 and MKN45 cells. This finding may indicate that mtDNA depletion associated with increase in chemoresistance may be a reflection of an association with CD44 positivity in HGC27 and MKN-45 metastatic GC cell lines. On the other hand, ρ^low^ AGS cells had increased chemoresistance in spite of decreased CD44 positivity. This finding may indicate that the association of CD44 positivity with the level of chemoresistance is only a metastasis signature and therefore absent in the non-metastatic AGS GC cell line. Further studies are needed to test and validate this hypothesis. 

In contrast to CD44, cell surface positivity for antiCD24 antibody decreased with mtDNA depletion in HGC-27 and MKN-45 cells. In addition, a decrease in chemoresistance was correlated with increased CD24 positivity in reverted HGC-27 and MKN-45 cells in spite of high CD44 positivity. This finding suggests that the mtDNA depletion-related increase in chemoresistance of metastatic HGC-27 and MKN-45 cell lines may be inhibited by increased cell surface expression of CD24, an attribute which may be related with the apoptosisinducing characteristic of CD24 (8).

HGC-27 and MKN-45, unlike AGS,partially maintained chemoresistance after reverting to normal mtDNA levels. The cell surface positivity was also found to be very low in AGS reverted cells. Given that CD44 is thought to be a chemoresistance inducer (24-26), the maintenance of chemoresistance after reversion may be associated with the level of CD44 positivity in reverted HGC-27 and MKN-45 cells.

## Conclusion

We not only confirm that mtDNA depletion triggers chemoresistance in correlation with an increase and decrease in CD44 and CD24 positivity respectively in HGC-27 and MKN-45 metastatic GC cell lines, but also, importantly, identified a mtDNA setpoint, at 33-40% of that observed in control cells, resulting in the highest levels of cell surface positivity for anti-CD44 antibody and chemoresistance. This mtDNA setpoint may thus be potentially used as a target for metastatic GC therapy if further independent studies are validated. 
